# Renoprotective effect of nicorandil in patients undergoing percutaneous coronary intervention: a meta-analysis of 4 randomized controlled trials

**DOI:** 10.18632/oncotarget.23965

**Published:** 2018-01-04

**Authors:** Xiaobing Wang, Jin Geng, Hong Zhu, Changying Xing

**Affiliations:** ^1^ Department of Nephrology, The First Affiliated Hospital of Nanjing Medical University, Jiangsu Province Hospital, Nanjing, Jiangsu, China; ^2^ Department of Nephrology, Taizhou Second People’s Hospital, Taizhou, Jiangsu, China; ^3^ Department of Cardiology, Huai’an First People’s Hospital, Nanjing Medical University, Huai’an, Jiangsu, China; ^4^ Department of Pharmacy, Huai’an First People’s Hospital, Nanjing Medical University, Nanjing, Jiangsu, China

**Keywords:** nicorandil, contrast-induced nephropathy, renal function, PCI, meta-analysis

## Abstract

Many studies have evaluated the renoprotective effect of nicorandil in patients undergoing percutaneous coronary intervention (PCI), but the results are inconsistent. We therefore conducted this meta-analysis to evaluate the protective effect of nicorandil against contrast-induced nephropathy (CIN). We searched PubMed, Embase, the Cochrane Library, Web of Science, and clinical trials database. Studies compared the nicorandil (plus hydration) with hydration alone in patients receiving PCI were eligible. The primary outcome was the incidence of CIN. Four randomized controlled trials (RCTs) with 730 patients were included. All enrolled patients were with renal dysfunction or with moderate risk for CIN. Meta-analysis showed that nicorandil was associated with a decrease of CIN (odds ratio 0.33, 95% confidence interval [CI], 0.19~0.58, *p* < 0.001), without heterogeneity across the studies (I^2^ = 33.7%, *p* = 0.210). Moreover, nicorandil treatment could significantly reduce the level of serum creatinine, estimated glomerular filtration rate and cystatin C at 48 hours after procedures (standardized mean difference [SMD] −0.17, 95%CI −0.33~–0.01; SMD 0.29, 95% CI 0.11~0.48; SMD −0.17, 95%CI −0.33~–0.01, respectively). Nicorandil can reduce the incidence of CIN and result in favorable changes in renal function in patients undergoing PCI. More RCTs with large sample size and high quality are needed to confirm our results.

## INTRODUCTION

With the increasing use of interventional procedures with contrast media, contrast-induced nephropathy (CIN) has become the third common cause of hospital-acquired acute kidney injury [1]. CIN accounts for 11%–12% of acute kidney injury in hospitalized patients, and the incidence is as high as 50% in high risk population [1–3]. It results in an increased risk of morbidity and mortality, prolonged hospitalization, and new onset of renal failure [3–5]. Use of iso- or low-osmolar contrast media and minimization of the media volume are the recommended nonpharmacologic approaches for preventing CIN [2, 6]. Besides, many pharmacologic strategies, such as hydration, statin, bicarbonate sodium, fenoldopam, natriuretic peptide, N-acetylcysteine, vitamins, theophylline and prostaglandin, have shown preventive effect against CIN [7].

Nicorandil is a hybrid compound derived from an ATP-sensitive K channel activator and a nitric oxide donor [8]. Nicorandil has been found to exert a cardiac preconditioning effect that improves microvascular circulation, leading to perioperative myocardial protective effect in patients undergoing percutaneous coronary intervention (PCI) [9]. Moreover, K-ATP channel opener ameliorates the renal reperfusion injury by preventing reactive oxygen species (ROS) accumulation [10]. Therefore, nicorandil may have renoprotective effect in patients receiving interventional procedures. Recently, many researchers evaluated the protective effect of nicorandil against CIN but provided inconsistent results [11–14]. We therefore conducted this meta-analysis to assess the efficacy of nicorandil for preventing CIN in high risk patients undergoing PCI.

## RESULTS

### Studies characteristics

**Figure 1:**
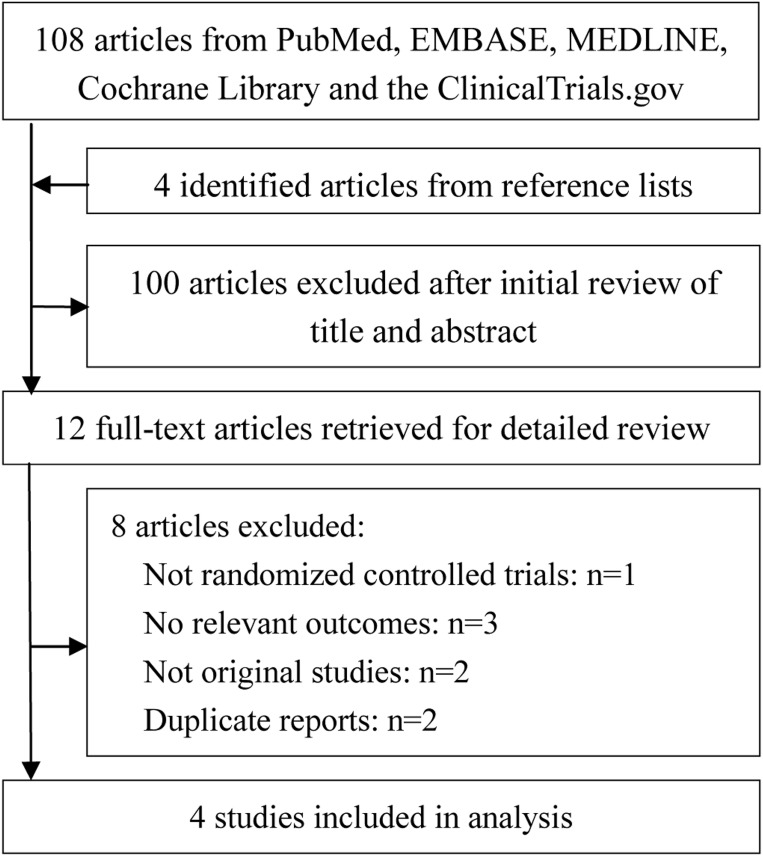
Flow diagram of selected studies for this meta-analysis

Figure 1 presents the flow diagram for study selection. In total, four randomized controlled trials (RCTs) involving 730 patients undergoing PCI were included [11–14]. Among these patients, 363 patients were assigned to the nicorandil group, and 367 patients were assigned to the control group. All patients were with moderate risk (defined by Mehran risk score) of developing CIN [14], or with poor renal function [11–13]. Nicorandil was administrated intravenously in two studies [11, 12], and orally in another two studies [13, 14]. Detailed characteristics of eligible studies are shown in Table 1.

**Table 1:** Characteristics of included studies

Author	Year	Country	Enrollment criteria	Nicorandil treatment	Hydration protocol	Contrst (volume, ml)	No.	Outcomes
Ko, Y. G	2013	Korea	eGFR≦60 ml/min and Scr ≧1.1 mg/dL	Intravenously, 12 mg, dissolved in 100 mL 0.9% saline	0.45% saline (1 mL/kg/h, 0.5 mL/kg/h for patients with LVEF < 40%)	Iodixanol (125.6 vs 126.9)	149	CIN, Scr, Cystatin C
Nawa, T	2015	Japan	Cystatin C > 0.95 mg/L (males) and 0.87 mg/dL (females)	Intravenously, 96 mg, dissolved in 100 mL saline (0.1ml/kg/h)	0.9% saline (1 mL/kg/h)	Iomeprol or iohexol (135.2 vs 146.3)	213	CIN, Scr^*^, eGFR^*^, Cystatin C^*^
Fan, Y	2016	China	eGFR < 60 ml/min	Oral, 30 mg/d, from 2d before to 3d after the procedure	0.9 % saline (1 mL/kg/h, 0.5 mL/kg/h for patients with LVEF < 40 %)	Ultravist (145.3 vs 149.2)	240	CIN, Scr, eGFR, Cystatin C
Iranirad, L	2017	Iran	moderate risk for CIN as defined by Mehran risk score	Oral, 10 mg/d, from 30 min before to 3d after the procedure	normal saline (1 mL/kg/h)	Iohexol (213.98 vs 202.26)	128	CIN, Scr, eGFR

^*^ Outcomes were expressed as percent change from baseline. eGFR, estimated glomerular filtration rate; Scr, serum creatinine; LVEF, left ventricular ejection fraction; CIN, contrast-induced nephropathy.

### Risk of bias assessment

**Figure 2:**
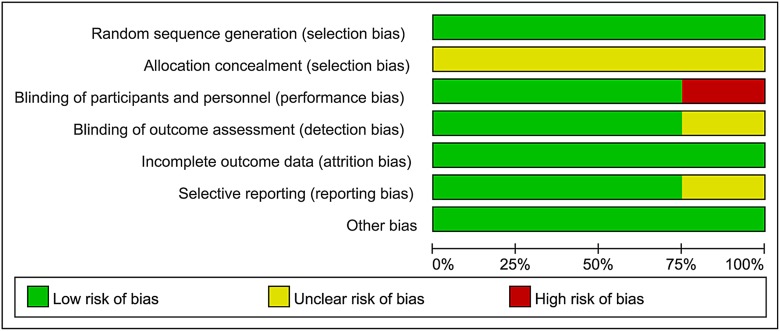
Assessment of the risk bias: bias of risk graph

**Figure 3:**
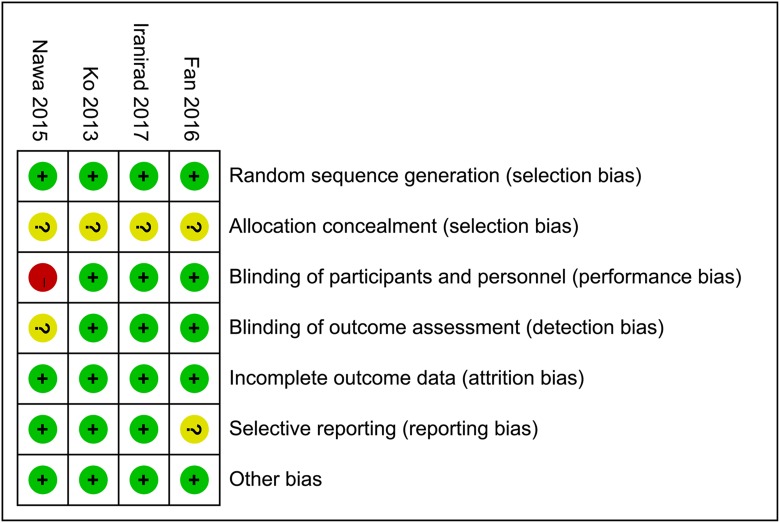
Assessment of the risk bias: bias of risk summary

All four studies generated allocation sequence and addressed incomplete outcome data adequately, but provided no relevant information of allocation concealment. One study by Nawa et al was not blinded [12]. Three studies provided registered information and were considered as low risk of reporting bias [11, 12, 14]. No other bias in each study was indentified. Quality assessment of eligible studies is available in Figures 2 and 3.

### Contrast-induced nephropathy

**Figure 4:**
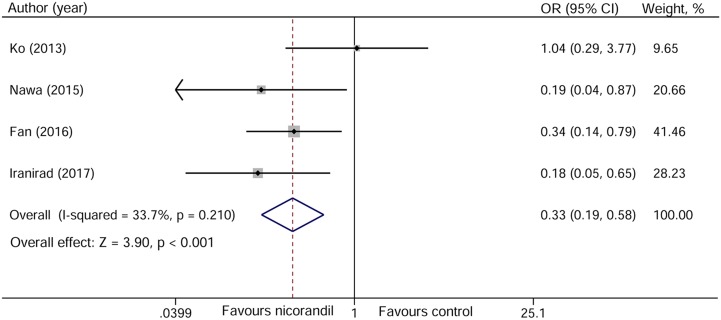
Forest plot of the incidence of contrast-induced nephropathy

**Figure 5:**
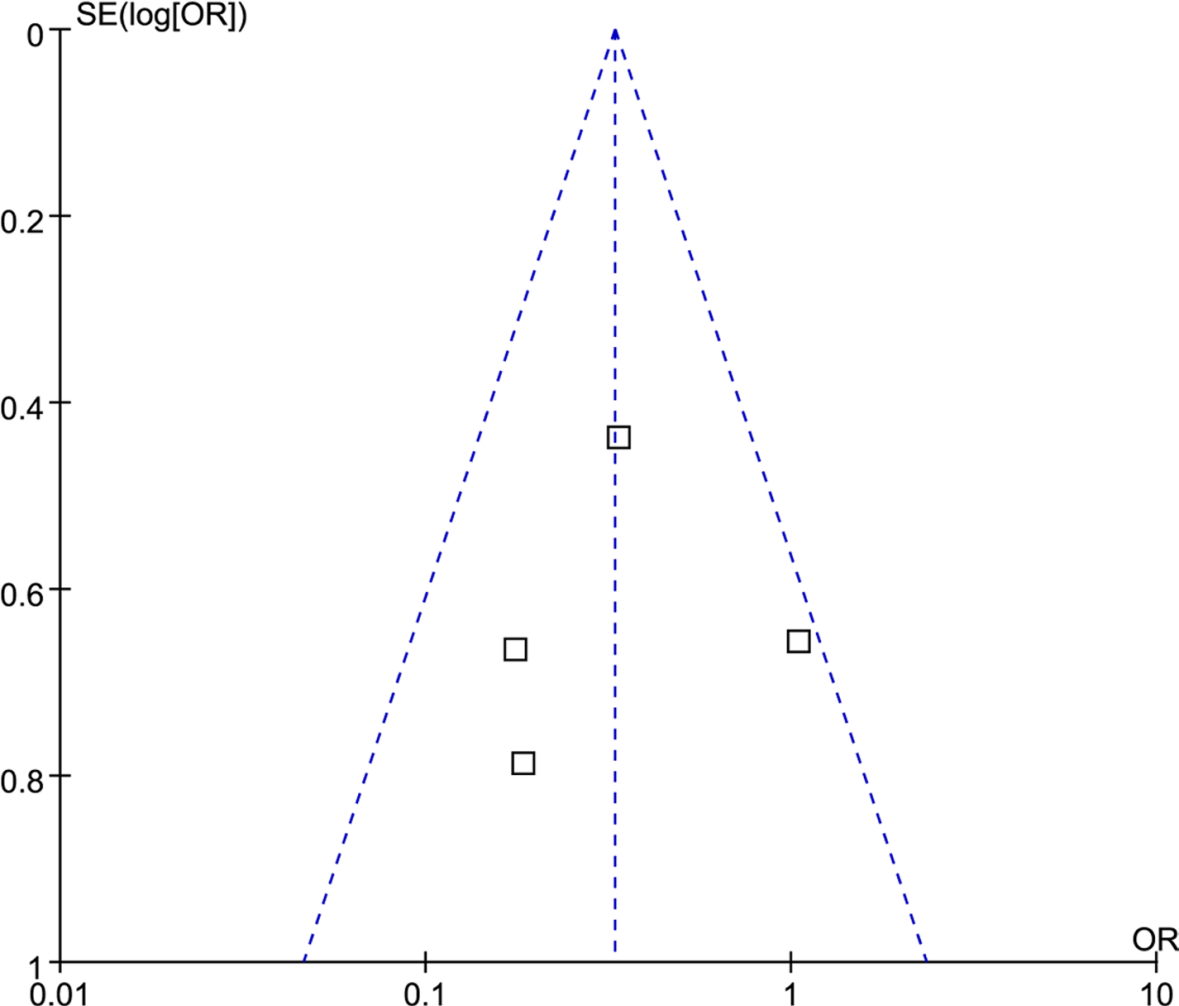
Funnel plot of the incidence of contrast-induced nephropathy

All four studies reported CIN data. The definition of CIN was consistent in these studies. Meta-analysis showed that nicorandil plus hydration significantly decreased the risk of CIN compared with hydration (odds ratio [OR] 0.33, 95% confidence interval [CI] 0.19~0.58, *p* < 0.001), with no evidence of heterogeneity across the studies (I^2^ = 33.7%, *P* = 0.210, Figure 4). No significant change of pooled estimate effect and heterogeneity were found after sensitivity analyses. Egger’s test revealed no statistical significant (*p* = 0.905) and the funnel plot seemed to be symmetric (Figure 5), indicating no potential publication bias.

### Serum creatinine

**Figure 6:**
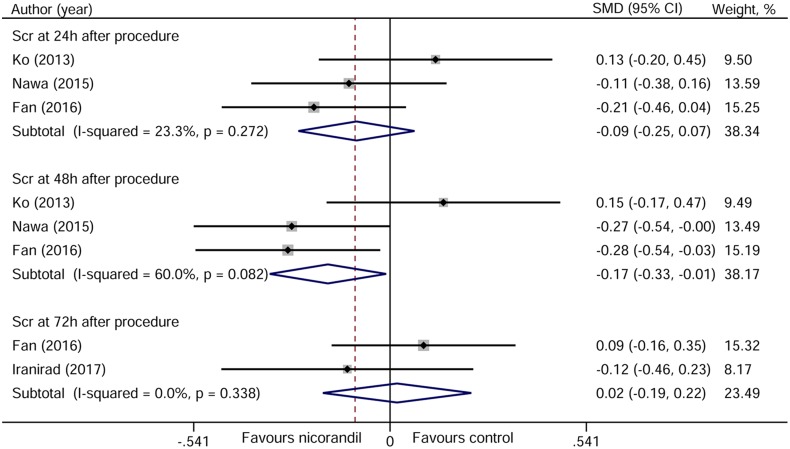
Forest plot of serum creatinine

Serum creatinine (Scr) data at 24 and 48 hours after PCI were provided in three studies [11–13], and data at 72 hours were provided in two studies [13, 14]. As shown in Figure 6, nicorandil treatment can significantly reduce the level of Scr at 48 hours after PCI (standardized mean difference [SMD] −0.17, 95% CI −0.33~–0.01, *p* = 0.037), but not at 24 hours (SMD −0.09, 95% CI −0.25~0.07, *p* = 0.257) and 72 hours (SMD 0.02, 95% CI −0.19~0.22, *p* = 0.853) after PCI.

### Estimated glomerular filtration rate

**Figure 7:**
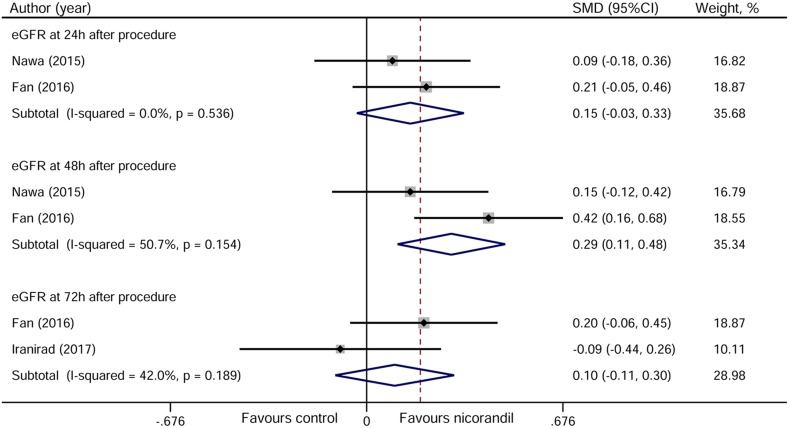
Forest plot of estimated glomerular filtration rate

Two studies reported data on estimated glomerular filtration rate (eGFR) at 24 and 48 hours after PCI [12, 13], and two reported data at 72 hours [13, 14]. As shown in Figure 7, nicorandil significantly increased the level of eGFR at 48 hours after PCI (SMD 0.29, 95% CI 0.11~0.48, *p* = 0.002), but not at 24 (SMD 0.15, 95%CI −0.03~0.33, *p* = 0.110) and 72 hours (SMD 0.10, 95%CI −0.11~0.30, *p* = 0.355) after PCI.

### Cystatin C

**Figure 8:**
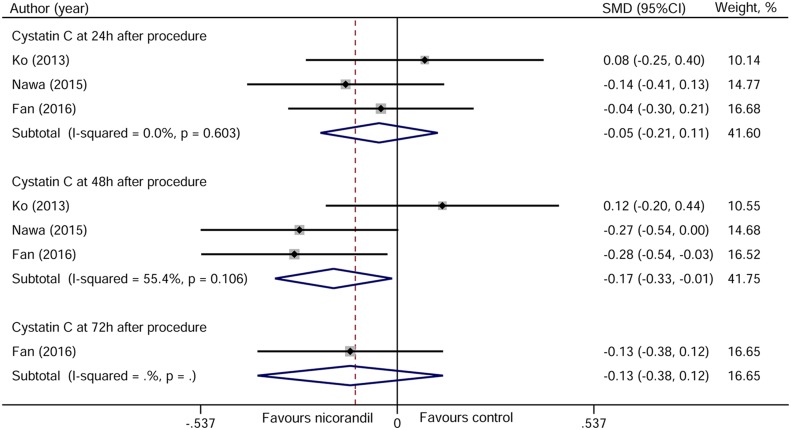
Forest plot of cystatin C

Three studies contributed to the pooled analysis of cystatin C at 24 and 48 hours after PCI [11–13], and only one study provided data on cystatin C at 72 hours after PCI [13]. Similar to the results of Scr and eGFR, cystatin C at 28 hours after PCI was significantly decreased in nicorandil group than in control group (SMD −0.17, 95%CI −0.33~–0.01, *p* = 0.033). No change of cystatin C level at 24 and 48 hours after PCI was found according to the meta-analysis (Figure 8).

## DISCUSSION

In the present study, we demonstrated that nicorandil treatment could decrease the risk of developing CIN in high risk patients undergoing PCI. Besides, renal function significantly improved in nicorandil group at 48 hours after PCI, but had no change at 24 and 72 hours. These data indicated that nicorandil is an alternative approach to prevent CIN in high risk population.

The underlying pathogenesis of CIN is not fully understood. It is likely that multiple mechanisms involving in the CIN. It is currently reported that major mechanisms are the direct toxicity on tubular cells, ischemic and hypoxic injury, and ROS accumulation [3, 15, 16]. Thus, medicines that have anti-inflammatory and inhibitory effect on ROS formation may be reasonable for CIN treatment [13]. Nicorandil is an ATP-sensitive K channel opener containing a nitric oxide donor, and has been found to have vasodilatory effects on the small vessels [8]. It has been reported to reduce chest pain, slow reflow phenomenon and arrhythmia caused by PCI [17, 18]. Meta-analysis of 16 RCTs confirmed the myocardial protective effect of nicorandil in patients receiving PCI [9]. The kidney is a vascular organ, and nicorandil is recently reported to have a renoprotective effect through suppression of ROS formation and improvement of blood flow [10]. Besides, nicorandil also protects the proximal tubule against ischemic reperfusion injury via the opening of K-ATP channels [19]. Also, nicorandil administration could improve endothelial function [20]. These results indicate that nicorandil may be an effective strategy for CIN treatment.

Ko et al firstly evaluated the preventive effect of nicorandil on CIN in patients with renal dysfunction undergoing PCI [11]. However, no statistical difference of the incidence of CIN was found between groups. Recently, three RCTs also compared the renoprotective effect of nicorandil with that of hydration in this population and found that nicorandil prevents CIN in patients with poor renal function experiencing PCI [12–14]. The inconsistent conclusion may be the result of difference in nicorandil dose. Patients in PRINCIPLE study were treated with 12 mg nicorandil prior to PCI [11], while in other three study, the minimal dose of nicorandil is 40 mg (Table 1). Besides, nicorandil was diluted in 100 ml saline and administrated intravenously in PRINCIPLE study [11]; however, nicorandil was continuous intravenous infused for more than 24 hours in Nawa’s study [12], and was administrated orally for several days in the other two studies [13, 14]. Another reason might be the different contrast agent. In the PRINCIPLE study, iso-osmolar contrast was used [11]; whereas low-osmolar agent was used in the other three studies [12–14].

Park et al also assessed the renoprotective effect of nicorandil in a retrospective design [21]. A total of 1492 patients with Scr less than 3.0 mg/dL were included, and no change in the CIN incidence between nicorandil group and control group was found. In contrast, only patients with renal dysfunction or moderate risk for CIN were enrolled in the studies included for our meta-analysis. Interestingly, iso-osmolar contrast agent was also used in Park’s study. It has been reported that iso-osmolar contrast media can significantly reduce the incidence of CIN when compared with low-osmolar contrast media [2]. And the incidence of CIN is 6.6% in control group from the PRINCIPLE study [11], which is lower than that in the other three studies [12–14]. Therefore, we hypothesized that nicorandil might be effective for preventing CIN only in patients with high risks of developing CIN. However, more studies are warranted to determine the dose and method of nicorandil delivery and to identify the population that could benefit from nicorandil.

Our meta-analysis of these four RCTs showed that nicorandil plus hydration was associated with a 67% decrease in the incidence of CIN compared with hydration in patients with renal insufficiency undergoing PCI. Many other pharmacologic strategies also have preventive effect on CIN, including statin, bicarbonate sodium, fenoldopam, natriuretic peptide, N-acetylcysteine, vitamins, theophylline and prostaglandin [7]. Among these approaches, high-dose statin with or without N-acetylcysteine plus hydration may be the preferred approaches and may prevent approximately 65% of CIN according the results of a network meta-analysis [7], which is similar to our results. However, further studies are needed to compare the efficacy of nicorandil with high-dose statin and N-acetylcysteine in patients at risk of developing CIN.

Several limitations must be acknowledged. First, only four RCTs with 730 patients were enrolled for the pooled analysis. Second, the dose and using time of nicorandil, as well as the method of drug delivery are not unified. Third, all studies were performed in Asia countries, lacking of data from Europe and North America. Fourth, although the articles included were all RCTs, one study was not blinded [12], and one study did not provided registered information [13]. There will be inevitable bias. Finally, we found that nicorandil treatment could improve renal function at 48 hours after PCI, but not at 24 and 72 hours. However, we can not clarify the underlying mechanism based on the available information.

In conclusion, our results suggest that nicorandil has a favorable preventive effect against CIN in patients with renal dysfunction. However, due to methodological limitations, the conclusion should be interpreted with caution. More RCTs with large sample size and high quality are needed to confirm the renoprotective effect of nicorandil.

## MATERIALS AND METHODS

The present study was conducted according to PRISMA guidelines [22], and the Cochrane Handbook for Systematic Reviews of Interventions [23], following a registered protocol on the PROSPERO database (CRD42017070005).

### Data sources and search strategy

We comprehensively searched PubMed, Embase, the Cochrane Library, Web of Science, and clinical trials database from the inceptions to June, 2017. Relevant keywords related to nicorandil (“Nicorandil” or “2-Nicotinamidoethyl Nitrate” or “2 Nicotinamidoethyl Nitrate” or “Nitrate, 2-Nicotinamidoethyl” or “2-Nicotinamidethyl Nitrate” or “2 Nicotinamidethyl Nitrate” or “Nitrate, 2-Nicotinamidethyl”) were used in combination with words related to CIN (“renal failure” or “kidney failure” or “kidney injury” or “CIN” or “renal insufficiency”). There was no language restriction and publication status. We also manually reviewed references of the identified articles and relevant reviews.

### Study selection

The inclusion criteria were described in accordance with PICOS acronym (participant, intervention, comparison, outcomes of interest and study design). For participants (P), all patients experiencing interventional procedures were included in this study. For intervention (I) and comparison (C), all studies must investigate the comparative effect of nicorandil plus hydration versus hydration. For outcomes (O), our primary outcome was the incidence of CIN, and the secondary outcomes were serum creatinine (Scr), estimated glomerular filtration rate (eGFR) and cystatin C after PCI. For study design, only randomized controlled trials (RCTs) were considered. The exclusion criteria were as follows: observational studies and non-RCTs, studies without relevant outcomes, reviews, and comments.

### Data extraction

Two reviewers (X.W & J.G) independently assessed available studies. Any discrepancies were solved by discussion with a third author (C.X). The extracted data consisted of the follow items: the first author’s name, publication year, country, enrollment criteria, nicorandila strategy, hydration protocol, sample size, and outcomes. We contacted the authors for any missing or unclear data.

### Quality assessment

We assessed the quality of included studies in accordance with the Cochrane Handbook for Systematic Reviews of Interventions, which includes 7 items: randomization sequence generation, allocation concealment, blinding of participants and study personnel, blinding of outcome assessors, incomplete outcome data, selective reporting, and other biases. “High bias risk”, “unclear bias risk” or “low bias risk” was considered for each study according to the extracted information.

### Statistical analysis

We used Stata 12.0 software to evaluate the pooled effect of CIN with the odds ratio (OR) and 95% confidence interval (CI), and of Scr, eGFR and cystatin C with the standardized mean difference (SMD) and 95% CI. The χ^2^-base *Q* test with a *p* < 0.10 and the I^2^ test with an I^2^ > 50% suggest significant heterogeneity [24]. Fixed effect model (Mantel-Haenszel method) was used preferentially [25]; and the random effect model (DerSimonian and Larid method) was used instead if high heterogeneity was indentified [26]. We also performed sensitivity and subgroup analyses to evaluate the contribution of including studies for heterogeneity. Publication bias was estimated using funnel plot and Egger’s test [27, 28]. A 2-tailed *P* < 0.05 was considered as statistical significance.

## Abbreviations

CIN: Contrast-induced nephropathy
PCI: percutaneous coronary intervention
ROS: reactive oxygen species
RCT: randomized controlled trial
OR: odds ratio
CI: confidence interval
Scr: serum creatinine
SMD: standardized mean difference
eGFR: estimated glomerular filtration rate
